# Ultrasound Imaging of Quadriceps Tendon in a Recreational Golfer

**DOI:** 10.24908/pocus.v6i2.14626

**Published:** 2021-11-23

**Authors:** Shawn D Felton, Arie J Van Duijn

**Affiliations:** 1 Florida Gulf Coast University Fort Myers, FL, 33965 USA

**Keywords:** Quadriceps Tendon, Point of Care Ultrasound, Knee, Golf

## Abstract

The patient was a 69-year-old recreational golfer who injured his right . While walking between the 9th and 18th holes, he slipped on pine straw. Ultrasound images of the quadriceps tendon post-injury revealed a full-thickness tear of the Quadriceps tendon, Rectus Femoris and Vastus intermedius. The diagnosis was confirmed through MRI arthrogram imaging. The hypoechoic finding in the ultrasound exam demonstrated the imaging to be as precise in diagnosing a full thickness tear as the MRI. The patient underwent surgical repair of the Quadriceps Tendon and is currently progressing in rehabilitation.

## Case File

**Figure 1:**
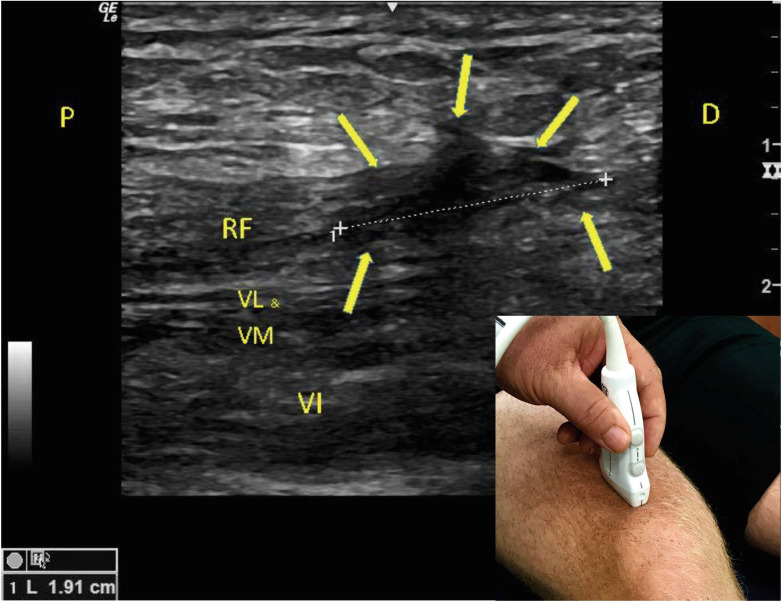
Post-injurylong-axis ultrasound image of the Rectus Femoris (RF) (5-12 MHz linear transducer). The proximal and distal aspect of the rectus femoris exhibits a normal hyperechoic appearance, but a substantial hypoechoic area is noted (arrowheads), 1.9 cm which is suggestive of fluid and a full thickness mid-tendon tear of the rectus femoris. Also, mild hypoechoic signaling indicating fluid in the medial and lateral muscles indicating a strain with mild hypoechoic signaling deep indicating strain of vastus intermedius. Positioning of the ultrasound probe is seen in the bottom right-hand corner of the image. Abbreviations: P: proximal; D: distal; RF: rectus femoris; VL: vastus lateralis; VM: vastus medialis; VI vastus intermedius.

**Figure 2:**
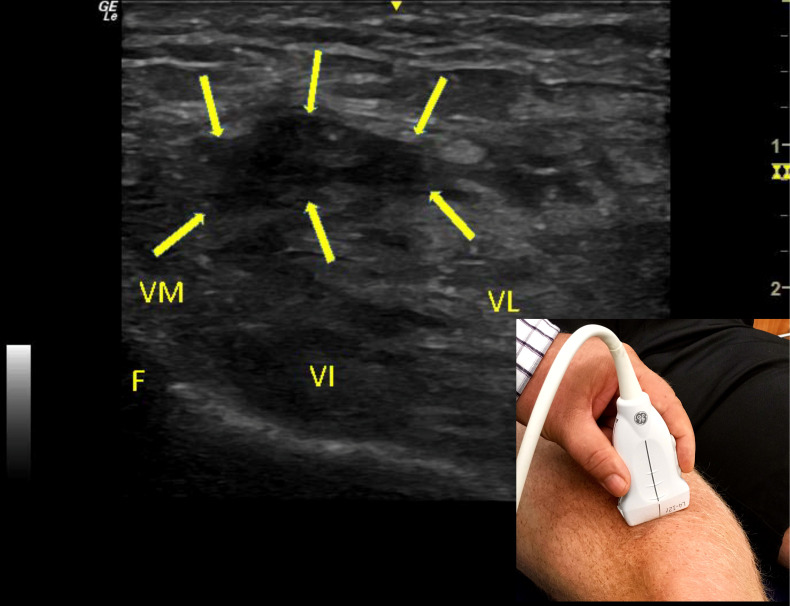
Post-injury short-axis ultrasound image of the Rectus Femoris. Disruption of the musculotendinous fibers of the rectus femoris can be visualized. A large hypoechoic gap in the rectus femoris is present, denoting the presence of significant fluid (arrows). Deeper areas of hypoechoic signaling indicating straining of the vastus medialis and lateralis and intermedius. Positioning of the ultrasound probe is seen in the bottom right-hand corner of the image. Abbreviations: F: femur; VL: vastus lateralis; VM: vastus medialis; VI vastus intermedius.

**Figure 3:**
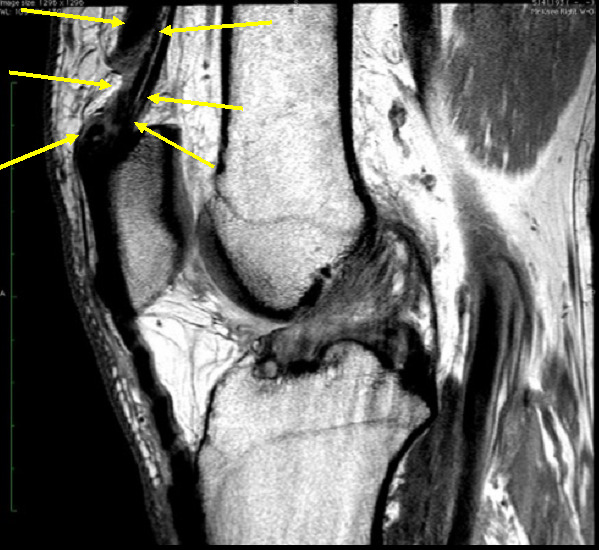
Post-injury MRI w/o contrast showing a portion of the quadriceps complex completely torn. The superficial aspect of the rectus femoris is avulsed from its patellar attachment and retracted proximally. Thinner smaller components of the vastus lateralis, vastus intermedius and vastus medialis remain intact. Arrowheads denote the torn quadriceps complex.

The patient was a 69-year-old recreational golfer who injured his right knee by slipping on pine straw, forcefully flexing his right knee under full weight-bearing. He indicated immediate sharp anterior knee pain and the inability to move his right leg. Upon examination, he presented with a palpable deformity proximal to the apex of the patella and moderate swelling at the lateral knee. The patient was unable to produce a quadriceps contraction and Passive Range of Motion (PROM) was limited due to pain. At time of injury, the patient had been prescribed three rounds of steroidal anti-inflammatory drugs for sinus infections, and had a prior history of a 3^rd ^degree quadriceps tear of his contralateral knee 12 years prior. Based upon these clinical findings, ultrasound imaging was performed at point-of-care showing full thickness mid-tendon tear of the rectus femoris (see Figures 1 & 2).The patient was referred to an orthopedic surgeon, who performed MRI imaging (see Figure 3), confirming an avulsion of the superficial aspect of the rectus femoris from its patellar attachment and retracted proximally 1.9 cm and a complete tear. Subsequently, the patient underwent immediate open repair of the quadriceps tendon and began rehabilitation after 6 weeks. 

This case illustrates the effectiveness of point of care ultrasound imaging identifying rectus femoris tendon tear. Prior researchhas indicated a high degree of clinical accuracy of ultrasound imaging for identifying partial and full thickness quadriceps tendon tears, comparable to MRI 1, 2. It is imperative for the clinician to ensure both short and long axis images to properly evaluate the extent of tissue damage. 

## Statement of Ethics

This study was approved by the Institutional Review Board of Florida Gulf Coast University

## Disclosures

The authors affirm they have no financial affiliation (including research funding) or involvement with any commercial organization that has a direct financial interest in any matter included in this manuscript, except as disclosed in an attachment and cited in the manuscript. 
